# Comparison of Herbal Medicines Used for Women’s Menstruation Diseases in Different Areas of the World

**DOI:** 10.3389/fphar.2021.751207

**Published:** 2022-02-04

**Authors:** Majing Jiao, Xinqiao Liu, Yongshen Ren, Yingzhou Wang, Long Cheng, Yunhui Liang, Yanqiu Li, Tianpei Zhang, Wen Wang, Zhinan Mei

**Affiliations:** School of Pharmacy, South-Central University for Nationalities, Wuhan, China

**Keywords:** herb medicine, ethnomedicine, premenstrual syndrome, dysmenorrhea, amenorrhea, menstrual disorders, women's health

## Abstract

**Aims:** This review aims to compare the use of herbal medicine used to treat women’s menstruation and the prevalence of menstrual diseases in different regions, which reveal the use of herbal medicine globally and provide scientific guidance for improving women’s health.

**Materials and Methods:** The information available on herbal medicines for women between the years 2000 and 2021 was systematically collected *via* the library and electronic search systems such as Google Scholar, PubMed, ScienceDirect, and Web of Science as well as secondary resources including books and conference proceedings.

**Results:** Totally, 571 ethnic medicines commonly used for women’s menstruation health in Asia, Europe, Oceania, Africa, and America were accounted. *Zingiber officinale* Roscoe (Ginger), *Ruta graveolens* L. (Common rue), *Angelica sinensis* (Oliv.) Diels (Angelica sinensis), *Foeniculum vulgare* Mill (Fennel), *Catharanthus roseus* (L.) G. Don (Catharanthus roseus) and other medicines which have obvious advantages and long-term usage are utilized in the treatment of menstrual diseases. Family Asteraceae, Lamiaceae, Apiaceae, Fabaceae, and Zingiberaceae are the most common medicinal plant families used for such treatments. In many instances, the application of fresh parts of plants was observed because of the healers’ belief regarding the higher efficiency of the medicine made from fresh plants. Edible plants are used in a wide range of countries.

**Conclusion:** Women’s menstruation health is directly related to their health condition. Traditional medicines of most ethnic groups have contributed to women’s health care and treatment of gynecological diseases. Practitioners in this field have gained elaborate experience in treatments and medication, and assembled a large number of effective drugs and prescriptions. These experiences have also been inherited and developed by modern clinical application and scientific research. However, the basic research on these drugs is not sufficient, the knowledge of drug use has not been fully popularized, the advantages of drugs have not been fully utilized, and the guiding potential to modern drug research continues to be insufficient. As such, it is necessary to further promote and make a significant contribution to women’s health.

## 1 Introduction

Herbal medicine has been widely used by women globally and increasingly more have shown the necessity of herbal medicines, both in treating diseases and maintaining health (Homaie Rad et al., 2021; Morehead and McInnis, 2021). Even in the modern world, there continue to be several health issues among women that cannot be cured using modern medicine; and in some regions having no modern medicines, it continues to be necessary to use herbal medicine as an alternative or supplement for women’s health and facilitate their life (Peltzer, 2009).

The menstrual pattern of a woman is indicative of her health status. Generally, menstruation occurs in regular intervals. Menstrual diseases (MD) include premenstrual syndrome (PMS), dysmenorrhea, amenorrhea, and menstrual disorders (MDs) (Kim, 2019). Ethnographic and epidemiological studies demonstrating significant variations in symptoms experienced by women with MDs living in different locales suggest that the expression of this biological event is mediated by many factors, including diet, lifestyle, cultural expectations and behaviors, and individual constitutions (Omani Samani et al., 2018). Hormonal therapy is the main treatment for MDs in modern medicine. However, considering the side effects of modern medicine, there are increasingly and more women who prefer to rely on herbal treatment based on their own traditional/national medicine.

Herbal medicines for women’s menstruation issues are used globally, and their efficacy and safety have been mentioned in previous reviews (Maleki-Saghooni et al., 2018). However, there continues to be a lack of references on the general character and differences for the administration of herbal medicine used by women all around the world. Therefore, this review aims to provide an overall blueprint on this theme by researching primary literature by gathering 130 families and 571 species of plants used by women from 22 countries and regions. The plants were categorized into four based on the types of diseases they treat. To the best of our knowledge, this is the first attempt to compile the list of herbal medicines used for women’s menstruation issues globally.

## 2 Methodology

### 2.1 Literature Search on Medicinal Plants for Menstrual Morbidity

We retrieved peer-reviewed scientific articles that documented traditional plant use in the study area related to MD. We followed the categories of menstrual morbidity defined by Harlow and Campbell (2004) and the definition of premenstrual syndrome (PMS) (Gnanasambanthan and Datta, 2019), and classified medicinal plants in four categories: 1) premenstrual syndrome; 2) dysmenorrhea; 3) amenorrhea; 4) menstrual disorders, including irregular cycles, such as a long duration of menstrual flow and excessive, heavy bleeding, (menorrhagia/polymenorrhagia) or delayed, infrequent menses (oligomenorrhea).

Literature reviews were performed during April-May 2021, using Google Scholar, PubMed, ScienceDirect, Web of Science and the keywords “menstruation,” “amenorrhea,” “arrested menstruation,” “obstructed menses,” “emmenagogue,” “dysmenorrhea,” “menses pain,” “menstrual pain,” “menstrual cramps,” “menstrual colic,” “excessive menses,” “excessive menstruation,” “menorrhagia,” “hypomenorrhea,” “oligomenorrhea,” “scanty menses,” “premenstrual syndrome,” “menstrual disorders,” “menses disturbance,” “abnormal menstruation,” “irregular menses” and “metrorrhagia” in combination with “herbs,” “medicinal plants” and “Africa,” “Asia,” “Latin America,” “Oceania,” “Europe” or specific countries in these geographical areas. Additional literature was found in reference lists of collected publications (snowball method). To limit the data to accessible digitized literature, we focused mostly on English literature published from 1980 onwards. Plant use information without a clear definition of the use, i.e., broad descriptions like “gynecological diseases” or “women’s health,” was not included in our review. Our geographical scope was restricted to countries or regions with detailed usage documents of medicinal plants. Our geographical review aimed to spread geographical coverage as much as possible over the continent, and used the most representative papers on this subject for each country/region (preferably the papers published in peer-reviewed journal and citing substantial ethnobotanical practices regarding our subject). The literatures cited in this article are evaluated according to the requirements of “the four pillars of best practice in ethnopharmacology (www.frontiersin.org/files/pdf/4_pillars_FULL_TEXT.pdf)”.

### 2.2 Methods of Screening Shortlisted Species

Relevant plants from the selected papers were entered in separate excel files for each of the five continents. Columns contained the four usage categories and rows represented the species. Individual cells contained the number of papers in which a particular use category for a particular species was reported. Scientific and author names were validated by the Plant List (http://www.theplantlist.org), and synonyms were merged. The most salient plants were defined as those most frequently cited in the literature (number of papers citing this species) and those mentioned for the most use categories. When describing the different countries and regions alphabetical order was applied in this paper, and Tables involving less than 20 kinds of medicinal plants are placed in the text, and tables involving more than 20 kinds of medicinal plants are provided as Supplementary Material.

## 3 Results

### 3.1 Ethnic Herbal Medicine Used for Premenstrual Syndrome

PMS is defined as a condition with emotional, physical, and behavioral symptoms that increase in severity during the luteal phase of the menstrual cycle and resolve by the end of menstruation. By definition, there must be a symptom-free interval after menstruation and before ovulation. Generally, symptoms are observed up to 14 days before the start of menses, causing impairment of life, with anger and irritability being the most severe and long lasting symptoms. The exact cause of PMS is unknown (Gnanasambanthan and Datta, 2019). However, studies have shown that 3–8% of menstruating women are affected by PMS and that 15–20% of women meet the criteria for subclinical PMS (O’Brien et al., 2011). A review reported incidences of PMS globally are 40, 85, 46, and 60% for Europe, Africa, Asia, and South America, respectively (Direkvand Moghadam et al., 2013).

The management of PMS is generally performed in a step-wise manner from non-pharmacological strategies, antidepressant medications, and hormonal strategies, with surgical options being considered as a last resort (Walsh et al., 2015). Studies have shown a more sustained but less rapid improvement with the use of selective serotonin reuptake inhibitors (SSRIs). Vitamin B6 is also well-known as the first-line treatment for PMS (Kashanian et al., 2007). However, peripheral neuropathy of pyridoxine is characterized at doses greater than 200 mg/day (Vrolijk et al., 2017). Therefore, herbs with lower costs, better benefits, and lesser side effects have become complementary and alternative treatments for women to improve PMS.

As a concept, PMS was put forward by western medicine doctors 70 years ago, and only a handful of herbal medicines are recorded in Western countries for the treatment (Gnanasambanthan and Datta, 2019). In Western countries, anti-depressive drugs, hormonal treatment, and analgesics dominate the treatment of PMS; and plant extracts such as *Vitex agnus-castus* L. is commonly considered as an alternative therapy in English-speaking countries (Weisz and Knaapen, 2009).

In China, the use of Chinese herbal medicine (CHM) is very common in the treatment of PMS. Liver qi stagnation and Yin Blood deficiency are proposed as the most common root causes of PMS, and the fundamental treatment principles for PMS involve measures to regulate Liver qi to resolve stagnation and tone blood (Chou and Morse, 2005). The most common medicines for the relief of PMS are Chaihu (*Bupleurum chinense* DC.), Xiangfu (*Cyperus rotundus* L.), Danggui (*Angelica sinensis* (Oliv.) Diels), Baishao (*Paeonia lactiflora* Pall.), and formulas such as Xiaoyao Powder and Jiawei Xiaoyao Powder (JXP). In Taiwan, JXP-centered CHM combinations were most commonly prescribed for PMS. The top 10 most commonly used single herbs for PMS are *Cyperus rotundus* L., *Leonurus japonicus* Houtt. (Oriental motherwort), *Corydalis yanhusuo* (Y. H. Chou and Chun C. Hsu) W. T. Wang ex Z. Y. Su and C. Y. Wu, *Salvia miltiorrhiza* Bge., *Eucommia ulmoides* Oliv., *Scutellaria baicalensis* Georgi, *Dipsacus asperoides* C. Y. Cheng at T. M. Ai, *Cuscuta chinensis* Lam. or *Cuscuta japonica* Choisy, *Pueraria lobata* (Willd.) Ohwi, *Paeonia × suffruticosa* Andrews. The top five most commonly used herbal formulas for the treatment of PMS are JXP, Danggui Shaoyao Powder, Guizhi Fuling Pills, Wenjing Decoction, and Shaofu Zhuyu Decoction (Chen et al., 2014). The ingredients of these Chinese herbal formulas are listed in Table 1.

**TABLE 1:** The ingredients of the Chinese herbal formulas.

Name	Number of ingredients	Ingredients	References
Danggui Shaoyao Powder	6	Paeoniae Radix Alba (*Paeonia lactiflora* Pall.), Chuanxiong Rhizoma (*Ligusticum chuanxiong* Hort.), Alismatis Rhizoma (*Alisma orientate*(Sam.)Juzep), Angelicae Sinensis Radix (*Angelica sinensis* (Oliv.) Diels), Poria (*Poria cocos* (Schw.) Wolf), and Atractylodis Rhizoma alba. (*Atractylodes macrocephala* Koidz.)	Lee HW et al. (2016)
Guizhi Fuling Pills	5	*Cinnamomi Cortex (Cinnamomum cassia Presl)*, Poria (*Poria cocos*(Schw.)Wolf), Moutan Cortex (*Paeonia suffruticosa* Andr.), Paeoniae Radix Alba (*Paeonia lactiflora* Pall.), and Persicae Semen (*Prunus persica* (L.) Batsch)	Sun et al. (2016)
GeGen Decoction	7	Puerariae Lobatae Radix(*Pueraria lobata*(Willd.)Ohwi), Ephedrae Herba (*Ephedra sinica* Stapf), Cinnamomi Cortex (*Cinnamomum cassia* Presl), Paeoniae Radix Alba (*Paeonia lactiflora* Pall.), Glycyrrhizae Radix Et Rhizoma (*Glycyrrhiza uralensis* Fisch.), Rhizoma Zingiberis recens (*Zingiber officinale* Roscoe) and Jujubae Fructus (*Ziziphus jujuba* Mil.)	Chai et al. (2020)
Jiawei Xiaoyao Powder	9	Bupleuri Radix (*Bupleurum chinense* DC.), Angelicae Sinensis Radix (*Angelica sinensis* (Oliv.) Diels), Paeoniae Radix Alba (*Paeonia lactiflora* Pall.), Atractylodis Rhizoma (*Atractylodes macrocephala* Koidz.), Smilacis Glabrae Rhizoma (*Smilax glabra* Roxb.), Glycyrrhizae Radix Et Rhizoma (*Glycyrrhiza uralensis* Fisch.), Moutan Cortex (*Paeonia suffruticosa* Andr.), Gardeniae Fructus (*Gardenia jasminoides* Ellis), Menthae Haplocalycis Herba (*Mentha canadensis* L.)	Li et al. (2019)
Shaofu Zhuyu Decoction	10	Foeniculi Fructus (*Foeniculum vulgare* Mill.), Rhizoma Zingiberis recens (*Zingiber officinale* Roscoe), Cinnamomi Cortex (*Cinnamomum cassia* Presl), Paeoniae Radix Alba (*Paeonia lactiflora* Pall.), Angelicae Sinensis Radix (*Angelica sinensis* (Oliv.) Diels), Chuanxiong Rhizoma (*Ligusticum chuanxiong* Hort.), Myrrh (*Commiphora myrrha* Engl.), Corydalis Rhizoma (*Corydalis yanhusuo* W. T. Wang), Typhae Pollen (*Typha angustifolia* L.), and Faeces Trogopterpri (*Trogopterus xanthipes* Milne Edwards)	Lee H et al. (2016)
Wenjing Decoction	12	Asini Corii Colla (*Equus asinus* L.), Ophiopogonis Radix (*Ophiopogon japonicus* (L. f) Ker-Gawl.), Pinelliae Rhizoma (*Pineilia ternata* (Thunb.) Breit.), Angelicae Sinensis Radix (*Angelica sinensis* (Oliv.) Diels), Glycyrrhizae Radix Et Rhizoma (*Glycyrrhiza uralensis* Fisch.), Cinnamomi Cortex (*Cinnamomum cassia* Presl), Paeoniae Radix Alba (*Paeonia lactiflora* Pall.), Chuanxiong Rhizoma (*Ligusticum chuanxiong* Hort.), Ginseng Radix Et Rhizoma (*Panax ginseng* C. A. Mey), Moutan Cortex (*Paeonia suffruticosa* Andr.), Euodiae Fructus (*Euodia rutaecarpa* (Juss.) Benth.) and Rhizoma Zingiberis recens (*Zingiber officinale* Roscoe)	Gao et al. (2017)
Xiaoyao Powder	8	Bupleuri Radix (*Bupleurum chinense* DC.), Radix Angelicae Sinensis Radix (*Angelica sinensis* (Oliv.) Diels), Paeoniae Radix Alba (*Paeonia lactiflora* Pall.), Atractylodis Rhizoma (*Atractylodes macrocephala* Koidz.), Poria (*Poria cocos* (Schw.) Wolf), Glycyrrhizae Radix Et Rhizoma (*Glycyrrhiza uralensis* Fisch.), Menthae Haplocalycis Herba (*Mentha canadensis* L.), and Rhizoma Zingiberis recens (*Zingiber officinale* Roscoe)	Liu et al. (2021)

In Iran, *Salvia officinalis* L. extract appears to be more effective in reducing the physical and psychological symptoms associated with PMS, when compared to placebo (Abdnezhad et al., 2019). *Vitex agnus-castus* L and *Hypericum perforatum* L. with lower doses of vitamin B6 are well tolerated and effective drugs to treat females with moderate to severe premenstrual syndrome in Iran (Ghazanfarpour et al., 2016). In Persian traditional medicine, saffron (dried stigma of *Crocus sativus* L.) is used for depression, which has been confirmed by modern medicine to be achieved through a serotonergic mechanism (Agha-Hosseini et al., 2008). A review about treatments in Iran has shown a reduction in PMS symptoms after consumption *Hypericum perforatum* L. (St. John’s wort), *Vitex agnus-castus* L. (Chasteberry), *Crocus sativus* L. (saffron), *Ginkgo biloba* L. (ginkgo), and soy (Golmakani et al., 2010). This is consistent with the data that we collected.

In Japan, Kampo medicine is the most preferred treatment choice for PMS. Kampo is a part of the official Japanese medical system and it is used alone or in combination with Western medicine for the treatment of complex health conditions, such as chronic health problems, age-related health problems, and lifestyle or stress-related disorders. We recorded 22 types of kampo’s that are the most commonly used ingredients (single herb) in PMS (Gepshtein et al., 2008) as shown in Table 2. Inochinohaha White is considered a medicine primarily used to treat PMS by attenuating anxiety-like behavior through GABAA receptor and brain-derived neurotrophic factor expression, which composed of 11 herbs: *Angelica sinensis* (Oliv.) Diels, *Paeonia lactiflora* Pall., *Atractylodes lancea* (Thunb.) DC., *Cinnamomum verum* J. Presl, *Rheum palmatum* L., *Panax ginseng* C. A. Meyer, *Cnidium monnieri* (L.) Cusson, *Poria cocos* (Schw.) Wolf, *Alisma plantago-aquatica* L., *Paeonia suffruticosa* Andr. and *Prunus persica* (L.) Batsch (Iba et al., 2021).

**TABLE 2:** Japanese herbal medicine for treating premenstrual syndrome.

Family	Scientific name	Used part	Habit
Lauraceae	*Cinnamomum cassia* (L.) J. Presl	Bark	Tree
Paeoniaceae	*Paeonia lactiflora* Pall.	Root	Herb
Rosaceae	*Prunus persica* (L.) Batsch	Seed	Tree
Paeoniaceae	*Paeonia × suffruticosa* Andrews	Bark	Shrub
Asteraceae/Compositae	*Atractylodes lancea* (Thunb.) DC.	Rhizome	Herb
Alismataceae	*Alisma orientalis* (Sam.) Juzep.	Rhizome	Herb
Polyporaceae	*Poria cocos* (Schw.) Wolf	Root	
Apiaceae	*Ligusticum chuanxiong* Hort.	Rhizome	Herb
Apiaceae	*Angelica acutiloba* var*. lineariloba* (Kitag.) Hikino	Root	Herb
Papaveraceae	*Corydalis yanhusuo* (Y. H. Chou and Chun C. Hsu) W. T. Wang ex Z. Y. Su and C. Y. Wu	Tuber	Herb
Ostreidae	*Ostrea gigas* thunberg	Root	
Fabaceae	*Glycyrrhiza uralensis* Fisch.	Root	Herb
Zingiberaceae	*Amomum villosum* var. *xanthioides* (Wall. ex Baker) T. L. Wu and S. J. Chen	Semen	Herb
Araliaceae	*Panax ginseng* C. A. Meyer	Root	Herb
Araceae	*Pinellia ternate* (Thunb.) Breit	Tuber	Herb
Rutaceae	*Aurantii Nobilis* Pericarpium	Peel	Tree
Zingiberaceae	*Zingiber officinale* Roscoe	Rhizome	Herb
Polyporaceae	*Polyporus umbellatus*		
Apiaceae	*Foeniculum vulgare* Mill.	Seed	Herb
Zingiberaceae	*Alpinia officinarum* Hance	Rhizome	Herb
Rhamniaceae	*Ziziphus jujuba* Mill.	Fruit	Tree

In Korea, herbal medicines for the treatment of PMS are *Hypericum perforatum* L. (St. John’s Wort), Odor of *Crocus sativus* L. (saffron), *Vitex agnus-castus* L. (Chasteberry), *Ginkgo biloba* L. (Ginkgo), *Cirsium japonicum* (Thunb.) Fisch. ex DC. (Cirsii Japonici Herba Carbonisata), *Elsholtzia splendens* Nakai ex F. Maekawa (Jang et al., 2012).

In South Africa, the pharmaceutical application of valerian (*Valeriana officinalis* L.) for the treatment of PMS is due to its sedative, anticonvulsant, hypnotic effects, and anxiolytic activity (Ghaderi and Jafari, 2014).

In summary, the most commonly used formula for the treatment of PMS in China is JXP. The most commonly used single drugs in the formulation are *Cyperus rotundus* L. and *Angelica sinensis* (Oliv.) Diels. In Western countries such as the United States, *Vitex agnus-castus* L. (chasteberry) and *Matricaria chamomilla* L. (chamomile) are regarded as the most available herbal alternative therapies.

### 3.2 Ethnic Herbal Medicine Used for Dysmenorrhea

Dysmenorrhea refers to the pain and swelling in the lower abdomen before and after menstruation or during menstruation, accompanied by backache or other discomfort (French, 2005). There are two types of dysmenorrhea: Primary dysmenorrhea refers to pain with no obvious pathological pelvic disease, whereas Secondary dysmenorrhea is caused by underlying pelvic conditions or pathology. Primary dysmenorrhea is considered to be caused by the release of prostaglandins in the menstrual fluid, which causes uterine contractions and pain. The reported prevalence of dysmenorrhea of any severity varies between 16 and 91% in women of reproductive age (Ju et al., 2014). Females who suffer from dysmenorrhea widely use non-steroidal anti-inflammatory drugs that reduce muscle spasm by inhibiting prostaglandin synthesis and vasopressin secretion (Sosorburam et al., 2019). In Western medicine, the main treatment trends tend to relieve prostaglandins synthesis and suppress their production from biosynthesis.

Native Americans commonly used whole plants of *Artemisia californica* Less. and fruits of *Rhus glabra* L. made a decoction to treat dysmenorrhea (Schmid and Moerman, 1998). Native American plants such as black haw (*Viburnum prunifolium* L.) and *Viburnum opulus* L. also utilized to treat painful menstruation (Lans et al., 2018).

According to the theories of CHM, the main cause of dysmenorrhea is Qi stasis caused by the invasion of the six exogenous pathogenic factors. Qi stasis results in the blockage of blood flow, which further leads to blood stasis and lumps. GeGen Decoction, Danggui Shaoyao Powder, and Guizhi Fuling Pillsare the famous Chinese prescriptions that are widely used in China to treat primary dysmenorrhea (Sosorburam et al., 2019). The ingredients of the Chinese herbal formulas are listed in Table 1. There are six types of Yao medicine used in treating dysmenorrhea, and among these three types of medicinal parts of plants are whole plants. Yao women are the best at using medicine baths to treat gynecological diseases, which is their ancient inheritance method (Long and Li, 2004; Li et al., 2006). As a famous herbal medicine used by Tujia and Miao Nationality, *Panax japonicus* (T. Nees) C. A. Mey is widely used for treating dysmenorrhea by local people (Deng et al., 2020). In Taiwan, Danggui Shaoyao Powder is the most frequently used formula that is prescribed by CHM doctors in Taiwan for treating menstrual cramps. The research shows that, among women with primary dysmenorrhea, CHM treatment is widely accepted by women in different ages, particularly those aged 21–30 years or those from lower income groups (Pan et al., 2014).

In India, a total of 38 types of herbs for the treatment of dysmenorrhea were collected from four pieces of literature (Jadhav and Bhutani, 2005; Vidyasagar and Prashantkumar, 2007; Bhatia et al., 2015; Das et al., 2015). Plants from the Asteraceae family are the main ones used to prepare dosages in the forms of decoction and infusion. Generally, they are administered orally. The leaf is the most important part of the plant that is used for medicinal purposes. Among them, 23 species are herbs, nine species are trees and two species are shrubs. *Vitex negundo* L. is the plant with the highest usage frequency. These four plants have been used in more than two countries: *Artemisia vulgaris* L., *Achyranthes aspera* L., *Matricaria chamomilla* L., and *Foeniculum vulgare* Mill. *Artemisia vulgaris* L. has been used to treat dysmenorrhea in India, Italy and Vietnam. Its multiple parts are effective in the treatment of dysmenorrhea.

In Italy, a total of 53 types of herbs were noted for the treatment of dysmenorrhea (Motti et al., 2019). The most common family of the plants are Asteraceae. The main forms of dosages are decoction and infusion. The most commonly used route of administration is oral administration. The most common medicinal parts are leaves. Among them, 37 species are herbs, eight species are trees, two species are shrubs and two species are vines. The herbal remedies mostly used for dysmenorrhea disorders are chamomile (*Matricaria chamomilla* L.), maidenhair fern (*Adiantum capillus-veneris* L.), yarrow (*Achillea millefolium* L.), and laurel (*Laurus nobilis* L.), which are taken orally, or as an infusion or decoction.

In South Africa, 30 types of plants are used to treat dysmenorrhea (Steenkamp, 2003). Roots are the medicinal parts of a majority of herbs. The most common dosage form is decoction. South Africa has a rich species of the herbs and most of them are local and unique species, such as *Commelina africana* L. Further, most of their names are related to the functional significance of the respective plants, such as *Pterocarpus angolensis* DC. (bloodwood), which is will used to treat dysmenorrhea, menorrhagia, and related diseases in South Africa. *Euclea crispa* Thunb. G˝urke is administered as enemas.

Herbs used in other countries to treat dysmenorrhea are listed in Table 3. See the Supplementary Material “Dysmenorrhea” for other specific contents.

**TABLE 3:** Herbal medicine for treating dysmenorrhea.

Country/Region	Family	Scientific name	Used part	Preparation	Application	Habit	References
America	Adoxaceae	*Viburnum prunifolium* L.	—	—	—	Shrub	Lans et al. (2018), Schmid and Moerman (1998)
America	Lauraceae	*Viburnum opulus* L.	—	—	—	Shrub	
America	Asteraceae	*Artemisia californica* Less.	Whole plant	Decoction		Herb	
America	Anacardiaceae	*Rhus glabra* L.	Fruit	Decoction		Shrub	
Brazil	Apiaceae	*Coriandrum sativum* L.	Seed	Infusion	—	Herb	Di Stasi et al. (2002), Bieski et al. (2015)
Brazil	Apiaceae	*Pimpinella anisum* L.	—	—	—	Herb	
Brazil	Aristolochiaceae	*Aristolochia cymbifera* Mart and Zucc	—	—	—	Shrub	
Brazil	Asteraceae	*Ageratum conyzoides* L.	Root	Infusion	—	Herb	
Brazil	Euphorbiaceae	*Jatropha curcas* L.	—	—	—	Shrub	
Brazil	Fabaceae	*Copaifera langsdorffii* Desf.	—	—	—	Tree	
Brazil	Lamiaceae	*Hyptis crenata* Pohl. Ex Benth.	Root	Infusion	—	Herb	
Brazil	Lamiaceae	*Mentha* x *piperita* L.	—	—	—	Herb	
Brazil	Lamiaceae	*Origanum majorana* L.	—	—	—	Herb	
Brazil	Lauraceae	*Cinnamomum verum* J. Presl	—	—	—	Tree	
Brazil	Lauraceae	*Cryptocarya mandioccana* Meisn.	—	—	—	Tree	
Brazil	Lauraceae	*Persea americana Mill*.	—	—	—	Tree	
Brazil	Myrtaceae	*Leptospermum scoparium* J. R. Forst. and G. Forst.	—	—	—	Shrub	
Brazil, India, Trinidad and, Tobago	Rutaceae	*Ruta graveolens* L.	Aerial part, whole plant (India), leaf (Brazil, India, Trinidad and Tobago)	Infusion (Brazil)	Orally	Herb	
Brazil, Trinidad, and Tobago	Lamiaceae	*Leonotis nepetifolia* (L.) R. Br.	—	—	—	Herb	
China	Apiaceae	*Angelica dahurica* (Fisch. ex Hoffm.) Benth. et Hook. f. ex Franch. et Sav	Root	—	—	Herb	Long and Li (2004), Li et al. (2006)
China	Araceae	*Acorus tatarinowii* Schott	Whole plant	Decoction or medicine bath	Topicaly	Herb	
China	Fabaceae	*Crotalaria ferrnginea* Grah. ex Benth	Whole plant	Decoction or medicine bath	Topicaly	Herb	
China	Lamiaceae	*Bryophyllum pinnatum* (L.F.) Oken	Whole plant	Decoction or medicine bath	Topicaly	Herb	
China	Lamiaceae	*Leonurus artemisia* (Laur.) S. Y. Hu F	Aerial part	—	—	Herb	
China	Ranunculaceae	*Clematis chrysocoma* Franch.	Woody stem	Decoction or medicine bath	Topicaly	Vine	
Iran	Apiaceae	*Echinophora platyloba* DC.	—	—	—	Herb	Mirabi et al. (2014)
Iran	Asteraceae	*Achillea willhemsii* L.	—	—	—	Herb	
Iran	Lamiaceae	*Stachys lavandulifolia* Vahl	—	—	—	Herb	
Iran	Lamiaceae	*Zataria multiflora* Boiss.	—	—	—	Herb	
Iran	Lauraceae	*Cinnamomum verum* J.Presl	—	—	—	Tree	
Iran	Rhamnaceae	*Cuminum cyminum* L.	—	—	—	Herb	
Iran	Valerianaceae	*Valeriana officinalis* L.	—	—	—	Herb	
Iran	Verbenaceae	*Vitex agnus-castus* L.	—	—	—	Shrub	
Iran	Asteraceae	*Matricaria chamomilla* L.	—	—	Orally	Herb	
Iran	Zingiberaceae	*Zingiber officinale* Roscoe	Rhizome	Powder	Orally	Herb	
Iran, Pakistan	Apiaceae	*Foeniculum vulgare* Mill.	Root, leaf (Iran, Pakistan), fruit (Iran, Pakistan)	—	Orally (Pakistan)	Herb	Ullah et al. (2014), Aziz et al. (2018), Tariq et al. (2018), Islam et al. (2021)
Pakistan	Asteraceae	*Achillea millefolium* L.	Leaf, root	Powder + water or milk	—	Herb	
Pakistan	Amaranthaceae	*Achyranthes aspera* L.	—	Decoction	Orally	Herb	
Pakistan	Poaceae/Gramineae	*Arundo donax* L.	Stem, leaf, fruit	—	—	Herb	
Pakistan	Asteraceae	*Erigeron bonariensis* L.	Whole plants, oil, shoot, leaf	Infusion	—	Herb	
Pakistan	Boraginaceae	*Arnebia hispidissima* (Lehm.) A.DC.	Whole plant	Diffusion and decoction	—	Herb	
Pakistan	Convolvulaceae	*Ipomoea eriocarpa* R. Br.	Leaf, Root	—	—	Shrub	
Pakistan	Cyperaceae	*Cyperus rotundus* L.	Whole plant (Pakistan), root (India), rhizome	Decoction	—	Herb	
Pakistan	Euphorbiaceae	*Euphorbia serpens* Kunth	Whole plant	—	Orally	Herb	
Pakistan	Moraceae	*Ficus benghalensis* L.	Latex, Bark, Fruit	—	—	Tree	
Pakistan	Nyctaginaceae	*Boerhaavia procumbens*	Whole plant	Juice	Orally	Herb	
Pakistan	Plantiginaceae	*Veronica agrestis* L.	Whole plant	Decoction	Orally	Herb	
Pakistan	Polygonaceae	*Persicaria amplexicaulis* (D. Don) Ronse Decr.	Flower, leaf, whole plant	—	—	Herb	
Poland	Asteraceae	*Ambrosia elatior* L.	Leaf	Infusion	—	Herb	Kujawska and Hilgert (2014)
Poland	Asteraceae	*Matricaria recutita* L.	Inflorescence, aerial part	Infusion, mate, poultice	—	Herb	
Poland	Asteraceae	*Tanacetum parthenium* (L.) Sch. Bip	Aerial part, leaf	Infusion	—	Herb	
Poland	Lamiaceae	*Cunila microcephala* Benth.	Leaf, stem	Infusion	—	Herb	
Poland	Lamiaceae	*Ocimum* sp.	Leaf, stem	Infusion	—	Herb	
Poland	Asteraceae	*Tanacetum vulgare* L.	Aerial part (Italy), leaf (Poland)	Infusion	Orally	Herb	
Poland	Poaceae/Gramineae	*Saccharum officinarum* L.	Leaf	Infusion, mate	—	Cane	
Poland	RaddiPteridaceae	*Hemionitis tomentosa* (Lam.)	Leaf, stem	Infusion	—	Herb	
Romania	Rosaceae	*Fragaria vesca* L.	Leaf	Infusion	—	Herb	Tita et al. (2009)
Romania	Araliaceae	*Hedera helix* L.	Leaf	Infusion	Orally	Vine	
Thailand	Araceae	*Acorus calamus* L.	Rhizome	—	—	Herb	Sitthithawor et al. (2019)
Thailand	Liliaceae	*Allium sativum* L.	Clove	—	—	Herb	
Thailand	Zingiberaceae	*Boesenbergia rotunda* (L.) Mansf.	Rhizome	—	—	Herb	
Thailand	Zingiberaceae	*Curcuma zedoaria* (Christm.) Roscoe	Rhizome	—	—	Herb	
Thailand	Myristicaceae	*Myristica fragrans* Houtt.	Seed	—	—	Tree	
Thailand	Zingiberaceae	*Zingiber montanum* (J. Koenig) Link ex A. Dietr.	Rhizome	—	—	Herb	
Trinidad and Tobago	Apiaceae	*Eryngium foetidum* L.	Leaf	—	—	Herb	Lans (2007)
Trinidad and Tobago	Aristolochiaceae	*Aristolochia trilobata* L.	Root	—	—	Herb	
Trinidad and Tobago	Asteraceae	*Ambrosia peruviana* Willd.	—	—	—	Herb	
Trinidad and Tobago	Boraginaceae	*Cordia curassavica* (Jacq.) Roem. and Schult.	Leaf	—	—	Shrub	
Trinidad and Tobago	Euphorbiaceae	*Croton gossypiifolius* Vahl	Leaf	—	—	Tree	
Trinidad and Tobago	Fabaceae	*Entada polystachya* (L.) DC.	Twigs	—	—	Vine, Shrub	
Trinidad and Tobago	Scrophulariaceae	*Capraria biflora* L.	Leaf	—	—	Herb	
Uganda	Aloaceae	*Aloe vera* L.	Leaf	Decoction	Orally	Herb	Kamatenesi-Mugisha and Oryem-Origa (2007)
Uganda	Amaranthaceae	*Aerva lanata* (L.) Schult.	Leaf	Decoction	Orally	Herb	
Uganda	Asteraceae	*Vernonia amygdalina* Del.	Leaf, root	Decoction	Orally	Shrub	
Uganda	Bignoniaceae	*Markhamia lutea* K. Schum.	Bark	Decoction	Orally	Tree	
Uganda	Caesalpiniaceae	*Cassia occidentalis* L.	Leaf, root	Decoction	Orally	Herb	
Uganda	Chenopodiaceae	*Chenopodium opulifolium* DC.	Leaf, bark, stem, seeds	Decoction	Orally	Herb	
Uganda	Convolvulaceae	*Ipomoea batatas* (L.) Lam.	Leaf, root tuber	Decoction	Orally	Herb	
Uganda	Euphorbiaceae	*Manihot esculenta* Crantz.	Leaf	Decoction	Orally	Shrub	
Uganda	Fabaceae	*Erythrina abyssinica* Lam.	Leaf, bark, flower	Decoction	Orally	Tree	
Uganda	Fabaceae	*Indigofera arrecta* A. Rich.	Leaf, root	Decoction	Orally	Shrub	
Uganda	Fabaceae	*Pseudarthria hookeri* Wight and Arn.	Leaf	Decoction	Orally	Shrub	
Uganda	Malvaceae	*Gossypium hirsutum* L.	Root	Decoction	Orally	Herb	
Uganda	Malvaceae	*Pavonia burchellii* (DC.) R.A. Dyer	Leaf	Decoction	Orally	Shrub	
Uganda	Moraceae	*Ficus natalensis* Hochst.	Aerial parts, leaf	Decoction	Orally	Tree	
Uganda	Myrtaceae	*Eucalyptus citriodora* Hook.	Leaf, fruit,gum	Decoction	Orally	Tree	
Uganda	Myrtaceae	*Eucalyptus globulus* Labill.	Leaf	Decoction	Orally	Tree	
Uganda	Myrtaceae	*Eucalyptus grandis* W.Hill.	Leaf	Decoction	Orally	Tree	
Uganda	Umbelliferae	*Daucus carota* L.	Root tuber	Decoction	Orally	Herb	
Uganda	Vitaceae	*Cyphostemma adenocaule* Wild and Drum.	Leaf, root	Decoction	Orally	Herb	
Uganda	Zingiberaceae	*Zingiber officinale* Roscoe	Root tuber	Decoction	Orally	Herb	
Vietnam	Asteraceae	*Artemisia vulgaris* L.	Fresh	—	—	Herb	Kurian (2012)
Vietnam	Apiaceae	*Centella asiatica* L.	Whole plant	—	—	Herb	

This section contains a total of 80 families and 217 species of plants from 16 countries and regions that are used for the treatment of dysmenorrhea. Asteraceae is the most commonly used among such species and there are five plants that have records of treating dysmenorrhea in three countries *Ruta graveolens* L. can treat dysmenorrhea and this is mentioned in two Brazilian documents. Italian women use its aerial parts to make decoctions, while women from Trinidad and Tobago use its leaves. Leaves, flowers, stems, and fruits of *Artemisia vulgaris* L. are used in India to treat dysmenorrhea and amenorrhea. The fresh whole plant is used by Vietnamese women, while Italian women use its aerial parts to make an infusion to treat dysmenorrhea. Considering *Cyperus rotundus* L., Indian women use its root and rhizome, while Pakistani women use the whole plant to make a decoction for oral administration and Yunnan Yao women in China use its root. Considering *Foeniculum vulgare* Mill. (Fennel), Pakistani women use its leaves and fruits orally; Iranian women use its roots, leaves, and fruits; Italian women use a decoction made from its seeds; and Chinese women use its dried and mature fruit. All three countries use the rhizome of *Zingiber officinale* Roscoe (ginger) to treat menstrual pain but the dosage forms are different: Malaysians make a lotion, Indians make a decoction, and Iranians make a powder.

### 3.3 Ethnic Herbal Medicine Used for Amenorrhoea

Amenorrhea (loss of menstrual period) is the absence of menstruation or absence of periods. There are two types of amenorrhea: Primary amenorrhea and Secondary amenorrhea. Primary amenorrhea can be diagnosed if a patient has normal secondary sexual characteristics but no menarche by 16 years of age. Secondary amenorrhea is the absence of menses for 3 months in women with previously normal menstruation and for 9 months in women with oligomenorrhea previously (Master-Hunter and Heiman, 2006). Secondary amenorrhea is more common than primary amenorrhea. The normal menstrual cycle involves a complex interaction between the hypothalamic-pituitary-ovarian axis; any disruption in this interaction can cause amenorrhea (Edmonds, 2007). Among women of reproductive age, the prevalence of amenorrhea ranged from approximately 5–13% (Harlow and Campbell, 2004). Hormonal therapy based on estrogen and progesterone compounds is the mainstay of the treatment for such conditions (Bergeron et al., 2010).

In native American herbs used to treat amenorrhea, the roots of *Acorus calamus* L. are made into infusion, while the rhizomes of *Acorus calamus* L. are used to treat dysmenorrhea in Thailand (Schmid and Moerman, 1998).

CHM often uses herbs that promote blood circulation, remove blood stasis, regulate menstruation and relieve pain to treat amenorrhea. Generally, these herbs can simultaneously treat dysmenorrhea. *Angelica sinensis* (Oliv.) Diels, *Leonurus japonicus* Houtt., and *Ligusticum chuanxiong* Hort. (Miao et al., 2019) are the three typical examples. *Curcuma longa* L. is the herbal medicine used by Chinese and Malaysians to treat amenorrhea. Barks and twigs of *Cinnamomum cassia* (L.) J. Presl both appear in Chinese and Japanese herbal medicines for the treatment of amenorrhea.

Japanese Herbal (Kampo) Medicine, which is covered by national health insurance in Japan, is often prescribed in the primary care field, and is also applied as an alternative remedy for several gynecological diseases, such as MDs and menopausal symptoms. Tokishakuyakusan (Chinese name, Dang gui shao yao san), Keishibukuryogan (Chinese name, Gui zhi fu ling wan), Kamishoyosan (Chinese name, Jia wei xiao yao san) and Unkeito (Chinese name, Wen jing tang) are most commonly used by Japanese women. *Paeonia lactiflora* Pall. is the most frequently ingredient contained in all the four herbal formulas (Kogure, 2011). The ingredients of the Japanese herbal formulas are listed in Table 4.

**TABLE 4:** Herbal medicine for treating amenorrhea.

Country/Region	Family	Scientific name	Used part	Preparation	Application	Habit	References
America	Acoraceae	*Acorus calamus* L.	Root	Infusion		Herb	Schmid and Moerman (1998)
America	Cannabaceae	*Celtis occidentalis* L.		Decoction		Tree	
America	Celastraceae	*Euonymus americanus* L.	Whole plant	Decoction		Shrub	
America	Fabaceae/Leguminosae	*Vicia sativa* subsp. nigra (L.) Ehrh.	Whole plant	Decoction		Herb	
America	Rhamnaceae	*Ceanothus americanus* L.	Root	Decoction		Shrub	
America	Rosaceae	*Malus coronaria* var. coronaria	Root	Decoction		Tree, Shrub	
America	Rubiaceae	*Mitchella repens* L.	Whole plant	Infusion		Herb	
Bangladesh	Amaranthaceae	*Amaranthus spinosus* L.	Root	Juice	Orally	Herb	Kadir et al. (2014)
Bangladesh	Apocynaceae	*Catharanthus roseus* (L.) G. Don.	Root	Juice	Orally	Subshrub	
Bangladesh	Euphorbiaceae	*Jatropha gossypifolia* L.	Leaf	Juice	Orally	Shrub, Tree	
Bangladesh	Lamiaceae	*Hyptis capitata* Jac q.	Root	Juice	Orally	Herb	
Bangladesh	Malvaceae	*Urena lobata* L.	Root	Juice	Orally	Subshrub	
Bangladesh	Moraceae	*Streblus asper* Lour.	Root	Juice	Orally	Shrub, Tree	
India	Apiaceae	*Daucus carota* L.	Seed	Tablet	Orally	Herb	Jadhav and Bhutani (2005), Vidyasagar and Prashantkumar (2007), Bhatia et al. (2015), Das et al. (2015)
India	Apiaceae	*Foeniculum vulgare* Mill.	Seed	Raw	Orally	Herb	
India	Aristolochiaceae	*Aristolochia bracteata* Ritz.	Leaf, root	—	—	Herb	
India	Brassicaceae	*Lepidium sativum* L.	Seed	Decoction	Orally	Herb	
India	Brassicaceae	*Raphanus sativus* L.	Seed	Powder	Orally	Herb	
India	Caesalpiniaceae	*Tamarindus indica* L.	Root, bark	Powder + Milk	Orally	Tree	
India	Caricaceae	*Carica papaya* L.	Unripe Fruit	Cooked Along With Ground Coconut Carnel, Green Chilly, Onion And Sufficient Quantity Of Salt	Orally	Herb	
India	Chenopodiaceae	*Chenopodium album* L.	Seed	Filtrate	Orally	Herb	
India	Fabaceae	*Trigonella foenum-graecum* L.	Whole plant	Raw	Orally	Herb	
India	Fabaceae	*Indigofera tinctoria* L.	—	Powder + Milk	Orally	Shrub	
India	Juglandaceae	*Juglans regia* L.	Fruit rind	Decoction	Orally	Tree	
India	Lamiaceae	*Ocimum tenuiflorum* L.	Leaf, Seed	Decoction	Orally	Herb	
India	Liliaceae	*Aloe vera* (L.) Burm.f.	Leaf	Gel	—	Herb	
India	Pedaliaceae	*Sesamum indicum* L.	Seed	—	—	Herb	
India	Poaceae/Gramineae	*Cynodon dactylon* (L.) Pers.	Whole plant	Mixed In Rice Soup	Orally	Herb	
India	Ranunculaceae	*Nigella sativa* L.	Seed	—	—	Herb	
India	Rutaceae	*Citrus limon* (L.) Burm. f.	Fruit	Juice	Orally	Tree	
India	Verbenaceae	*Vitex negundo* L.	Root	Cooked Along With Rice In The Form Of Porridge	Orally	Shrub	
Italy	Rutaceae	*Ruta graveolens* L.	Aerial part, Leaf	Decoction	Orally	Herb	Motti et al. (2019)
Japan	Apiaceae	*Angelica sinensis* (Oliv.) Diels^a^	Root	Powder	Orally	Herb	Kogure (2011)
Japan	Apiaceae	*Bupleurum scorzonerifolium* Willd.^a^	Root	Powder	Orally	Herb	
Japan	Asteraceae/Compositae	*Atractylodes lancea* (Thunb.) DC.^a^	Rhizome	Powder	Orally	Herb	
Japan	Fabaceae	*Glycyrrhiza uralensis* Fisch.^a^	Root	Powder	Orally	Herb	
Japan	Lamiaceae	*Mentha canadensis* L.^a^	Whole Plant	Powder	Orally	Herb	
Japan	Polyporaceae	*Poria cocos* (Schw.) Wolf^a^	Sclerotium	Powder	Orally		
Japan	Ranunculaceae	*Paeonia lactiflora* Pall.^a^	Root	Powder	Orally	Herb	
Japan	Ranunculaceae	*Paeonia suffruticosa* Andr.^a^	Bark	Powder	Orally	Shrub	
Japan	Rubiaceae	*Gardenia jasminoides* J. Ellis^a^	Fructus	Powder	Orally	Herb	
Japan	Zingiberaceae	*Zingiber officinale* Roscoe^a^	Rhizome	Powder	Orally	Herb	
Japan	Lauraceae	*Cinnamomum* cassia (L.) J. Presl^b^	Twig	Pill	Orally	Tree	
Japan	Lauraceae	*Cinnamomum cassia* (L.) J. Presl^b^	Bark	Pill	Orally	Tree	
Japan	Polyporaceae	*Poria cocos* (Schw.) Wolf^b^	Carpophores	Pill	Orally		
Japan	Ranunculaceae	*Paeonia lactiflora* Pall.^b^	Root	Pill	Orally	Herb	
Japan	Ranunculaceae	*Paeonia lactiflora* Pall.^b^	Root bark	Pill	Orally	Herb	
Japan	Rosaceae	*Prunus persica* (L.) Batsch^b^	Seed	Pill	Orally	Tree	
Japan	Alismataceae	*Alisma orientalis* (Sam.) Juzep.^c^	Tuber	Powder	Orally	Herb	
Japan	Apiaceae	*Angelica sinensis* (Oliv.) Diels^c^	Root	Powder	Orally	Herb	
Japan	Apiaceae	*Cnidium officinale* Makino^c^	Root stem	Powder	Orally	Herb	
Japan	Asteraceae/Compositae	*Atractylodes lancea* (Thunb.) DC.^c^	Rhizome	Powder	Orally	Herb	
Japan	Polyporaceae	*Poria cocos* (Schw.) Wolf^c^	Sclerotium	Powder	Orally		
Japan	Ranunculaceae	*Paeonia lactiflora* Pall.^c^	Root	Powder	Orally	Herb	
Japan	Apiaceae	*Angelica sinensis* (Oliv.) Diels^d^	Root	Decoction	Orally	Herb	
Japan	Apiaceae	*Cnidium officinale* Makino^d^	Rhizome	Decoction	Orally	Herb	
Japan	Araceae	*Pinellia ternate* (Thunb.) Breit.^d^	Tuber	Decoction	Orally	Shrub	
Japan	Araliaceae	*Panax ginseng* C. A. Meyer^d^	Radix	Decoction	Orally	Herb	
Japan	Equidae	*Equus asinus* L.^d^	Skin	Decoction	Orally		
Japan	Fabaceae	*Glycyrrhiza uralensis* Fisch.^d^	Root	Decoction	Orally	Herb	
Japan	Lauraceae	*Cinnamomum cassia* (L.) J. Presl^d^	Bark	Decoction	Orally	Tree	
Japan	Liliaceae	*Ophiopogon japonicus* (Linn. f.) Ker-GawL.^d^	Tuber	Decoction	Orally	Herb	
Japan	Ranunculaceae	*Paeonia lactiflora* Pall.^d^	Root	Decoction	Orally	Herb	
Japan	Ranunculaceae	*Paeonia suffruticosa* Andr.^d^	Bark	Decoction	Orally	Herb	
Japan	Rutaceae	*Tetradium ruticarpum* (A. Jussieu) T. G. Hartley^d^	Fruit	Decoction	Orally	Shrub, Tree	
Japan	Zingiberaceae	*Zingiber officinale* Roscoe^d^	Rhizome	Decoction	Orally	Herb	
Malaysia	Zingiberaceae	*Curcuma longa* L.	—	—	—	Herb	Ling and Ng (1998)
Malaysia	Zingiberaceae	*Curcuma xanthorrhiza* D. Dietr.	—	—	—	Herb	
Pakistan	Amaranthaceae	*Amaranthus spinosus* L.	Whole plant	Decoction	Orally	Herb	Tariq et al. (2018)
Pakistan	Asphodelaceae	*Aloe barbadensis* Mill.	Leaf	Powder + milk	Orally	Herb	
Pakistan	Bereridaceae	*Berberis lycium* Royle.	Root	Infusion	—	Shrub	
Pakistan	Bombacaceae	*Bombax ceiba* L.	Root,gum,flower	Powder + milk	Orally	Tree	
Pakistan	Lamiaceae	*Ajuga parviflora* Benth.	Leaf, Root	Powder + water	Orally	Herb	
Pakistan	Ulmaceae	*Celtis australis* L.	Fruit, Bark	Decoction + ghee	Orally	Tree	
Pakistan	Urticaceae	*Urtica dioica* L.	Whole plant	Powder + water	Orally	Herb	

Note:

a , Kamishoyosan (Chinese name, Jia wei xiao yao san).

b , Keishibukuryogan (Chinese name, Gui zhi fu ling wan).

c , Tokishakuyakusan (Chinese name, Dang gui shao yao san).

d , Unkeito (Chinese name, Wen jing tang).

Traditional Persian medicine (TPM), as a holistic system of medicine and based on temperament, has been used in Iran since thousands of years ago (Hosseinkhani et al., 2021). Temperament is made of action and reaction of four pivotal elements (fire, air, water, and soil) and creates different characteristics in living things. In TPM, temperament has been classified in different types: hot, cold, wet, and dry (Akhtari et al., 2020). Amenorrhea, oligomenorrhea, and hypomenorrhea are defined as “Ehtebas Tams” in TPM. From TPM viewpoint, anatomical and functional disorders (mal-temperaments) system are the main causes of oligomenorrhea and amenorrhea (Rahimi and Ardekani, 2013). The most prevalent temperaments of plants used to treat amenorrhea in Iranian medicine were warm and dry. *Foeniculum vulgare* Mill., *Mentha longifolia* (L.) L., *Paeonia lactiflora* Pall., *Sesamum indicum* L., and *Vitex agnus-castus* L. are the five most effective and documented herbs for treating amenorrhea (Moini Jazani et al., 2018).

In South Africa, plants belonging to 15 families are used for the treatment of amenorrhea. The most common plant families reported are the Fabaceae (five species) and Asteraceae (two species). *Asparagus buchananii* Baker is burnt and its smoke is directed into the vagina. *Boscia foetida* Schinz shows potential toxicity with hemorrhagic diarrhea. Powdered plant material is also applied to underwear: *Albizia brevifolia* Schinz, *Brackenridgea zanguebarica* Oliver, and *Pterocarpus angolensis* DC. are used for all for the treatment of amenorrhea (Steenkamp, 2003). The roots of *Rhoicissus digitata* (L. f.) Gilg and M. Brandt are chopped and mixed with the same number of chopped roots of *Bridelia catharti*ca G. Bertol. and *Peltophorum africanum* Sond. Four handfuls of the mixed plant material are boiled in 5 L of water for 1 h in a pot with a lid on. Half a cup of the decoction is taken orally two to three times a day (depending on one’s preference). This decoction treats amenorrhea (de Wet and Ngubane, 2014).

Herbs used in other countries and regions to treat amenorrhea are listed in Table 4. See the Supplementary Material “Amenorrhea” for other specific contents.

This part contains a total of 53 families and 99 species of plants from nine countries and regions used for the treatment of amenorrhea. Ranunculaceae family is the most commonly used among these species and some plants have records of treating amenorrhea in two countries: women use leaves of *Aloe barbadensis* Mill. in both India and Pakistan, but the dosage forms are different. Indian women make a gel for usage, while Pakistani women use its powder and milk. The whole plant of *Amaranthus spinosus* L. is made into a decoction in Pakistan and its roots are squeezed into a juice in Bangladesh.

### 3.4 Ethnic Herbal Medicine Used for Menstrual Disorders

MDs are some of the most common conditions to affect reproductive-aged women globally. They include abnormal menstrual cycle length, hypomenorrhea, and menorrhagia (Harlow et al., 2000). An epidemiological survey found that 27.2% of women suffered from MDs in five European countries (Fraser et al., 2015). Although estrogen therapy is beneficial in patients with MDs, its side effects such as nausea and vomiting had led to significant distress (Wang et al., 2020).

In America, *Artemisia dracunculus* L. (tarragon), whose whole plants are used for treating irregular menstruation by the Indians of the Missouri River Region, has long been used in traditional Asian medicine such as Myanmar while known as a spice species in Asia, Europe and the America (Ekiert et al., 2021). Native Americans use leaves of *Artemisia vulgaris* L. to treat irregular menstruation, while Indians, Italians and Vietnamese use it to treat dysmenorrhea (Schmid and Moerman, 1998).

The traditional Chinese medication experience and modern application of herbal medicine in treating MDs have enhanced people’s recognition of its importance and necessity. Dried products of herbal medicine are often used in traditional Chinese medicine which is convenient for transportation and storage. *Rehmannia glutinosa* (*Rehjnannia glutinosa* Libosch.) and its processed products play an important role in the treatment of irregular menstruation. Modern research shows (Zhang et al., 2008) that Chinese Rehmanniae Radix can prevent an inducement of the peripheral microcirculation of various chronic diseases through the improvement of hemorheology. Chinses angelica (*Angelica sinensis* (Oliv.) Diels) is a Chinese herbal medicine traditionally used for replenishing blood. Studies have shown that *Angelica sinensis* polysaccharide significantly reduced the apoptosis rate of platelets and had an anti-apoptosis effect on cryopreserved platelet (Nai et al., 2021).

In India, there are 73 species of plants used to treat MDs (Jadhav and Bhutani, 2005; Vidyasagar and Prashantkumar, 2007; Bhatia et al., 2015; Das et al., 2015). The major used family being Fabaceae; its leaves and roots are used often. Juice is the most frequently used method of administration, and oral administration is the most commonly used route of administration.

In Iran, a review suggests that the average prevalence of MDs is substantial (Omani Samani et al., 2018). Tansaz et al. (2015) summarized a variety of herbal medicines used for treating menorrhagia in Iran, including two forms, namely simple medicines and compound medicines. Plantain (*Plantago asiatica* L.) is one of the best medicinal plants used for menorrhagia treatment that is used by some of the traditional Iranian women. This plant is used either orally or vaginally. The other plant that is used frequently by Iranian traditional women is *Punica granatum* L., which grows in the wild in Iran, and it can be used either orally or topically. In the medical approach, traditional Iranian medicine physicians apply multiple drug dosage forms, such as oral, vaginal suppository, sitz bath, lotion, cleansing, and balm. These varieties increase a physician’s options in the management of menorrhagia and improve the compliance of patients.

In South Africa, Fabaceae is the most common family that appears in the treatment of menorrhagia among 31 plants (Steenkamp, 2003). *Adenia gummifera* (Harv.) Harms (Passifloraceae) and *Xylopia longipetala* De Wild. and T. Durand. (Annonaceae) are used as vaginal douches.

In Thailand, 25 species of plants are used to treat MDs (Sitthithawor et al., 2019). Zingiberaceae is the prominent family of species, while the primary part used is rhizome and oral administration is the major route of administration.

Herbs used in other countries and regions to treat MDs are listed in Table 5. See the Supplementary Material “Menstrual disorders” for other specific contents.

**TABLE 5:** Herbal medicine for treating menstrual disorders.

Country/Region	Diseases	Family	Scientific name	Used part	Application	Preparation	Habit	References
America	Irregular menstruation	Adoxaceae	*Viburnum lentago* L.	Root	Infusion		Tree	Schmid and Moerman (1998)
America	Irregular menstruation	Apiaceae	*Sanicula marilandica* L.	Bulb roots	Infusion		Herb	
America	Irregular menstruation	Asteraceae	*Artemisia dracunculus* L.	Whole Plant	Decoction	Orally	Subshrub	
America	Irregular menstruation	Asteraceae	*Artemisia frigida* Willd.	Whole Plant	Decoction	Topically	Herb	
America	Irregular menstruation	Fabaceae	*Tephrosia virginiana* (L.) Pers.	Whole Plant	Decoction	Topically	Herb	
America	Irregular menstruation	Liliaceae	*Lilium canadense* L.	Whole Plant	Infusion		Herb	
America	Irregular menstruation	Rosaceae	*Fragaria virginiana* Duchesne	Whole Plant	Infusion		Herb	
America	Irregular menstruation	Rosaceae	*Rubus pubescens* var. pubescens	Whole Plant	Infusion		Herb	
America	Menorrhagia	Asteraceae	*Artemisia douglasiana* Besser ex Besser	Leaf	Decoction, infusion	Orally	Herb	
America	Menorrhagia	Asteraceae	*Artemisia vulgaris* L.	Leaf	Decoction	Orally	Herb	
America	Menorrhagia	Convolvulaceae	*Convolvulus arvensis* L.	Stem with leaves	Decoction	Orally	Herb	
America	Menorrhagia	Ericaceae	*Pyrola chlorantha* Sw.	Whole Plant			Herb	
America	Menorrhagia	Pinaceae	*Pseudotsuga menziesii* (Mirbel) Franco	Green bark	Infusion	Orally	Tree	
America	Menorrhagia	Pteridaceae	*Adiantum pedatum* L.	Root	Decoction, infusion	Orally	Herb	
America	Menorrhagia	Ulmaceae	*Ulmus americana* L.	Root bark	Infusion	Orally	Tree	
America	Menstrual disorders	Apiaceae	*Osmorhiza occidentalis* (Nutt.) Torr.	Root	Decoction		Herb	
America	Menstrual disorders	Asparagaceae	*Maianthemum stellatum* (L.) Link	Root	Decoction		Herb	
America	Menstrual disorders	Asteraceae	*Artemisia ludoviciana* ssp. Ludoviciana	Leaf	Infusion		Herb	
America	Menstrual disorders	Caprifoliaceae	*Symphoricarpos albus* (L.) S.F.Blake	Branch	Infusion		Shrub	
America	Menstrual disorders	Cupressaceae	*Thuja occidentalis* L.	Whole Plant	Infusion		Tree	
Argentina	Menstrual disorders	Apiaceae	*Apium graveolens* L.	Leaf	Decoction	Orally	Herb	Hilgert (2001)
Argentina	Menstrual disorders	Lauraceae	*Cinnamomum verum* J.Presl	Whole plant	Decoction	Orally	Tree	
Argentina	Menstrual disorders	Pteridaceae	*Adiantopsis chlorophylla* (Sw.) Fée	Whole plant	Infusion + honey	Orally	Herb	
Argentina	Menstrual disorders	Pteridaceae	*Adiantum poiretii* Wikstr.	Whole plant	Infusion + honey	Orally	Herb	
Argentina	Menstrual disorders	Ranunculaceae	*Clematis haenkeana* C.Presl	Whole plant	Infusion + honey	Orally	Vine	
Argentina	Menstrual disorders	Rutaceae	*Ruta chalepensis* L.	Leaf	Infusion + honey	Orally	Herb	
Argentina	Menstrual disorders, menorrhagia	Lamiaceae	*Origanum vulgare* L.	Whole plant	soft Drink + honey	Orally	Subshrub, Herb	
Argentina	Menstrual hemorrhage	Asteraceae	*Anthemis cotula* L.	Whole plant	Infusion + honey	Orally	Herb	
Argentina	Menstrual hemorrhage	Piperaceae	*Peperomia fiebrigii* C. DC.	whole plant	Infusion + Honey	Orally	Herb	
Argentina	Menstrual hemorrhage	Scrophulariaceae	*Agalinis fiebrigii* (Diels) D’Arcy	Flower	Infusion	Orally	Shrub	
Bangladesh	Menorrhagia	Fabaceae	*Saraca asoca* (Roxb.) De Wilde	Dark	Decoction	Orally	Tree	Kadir et al. (2014)
Bangladesh, Myanmar	Menorrhagia	Plantaginaceae	*Scoparia dulcis* L.	Whole plant (Bangladesh), root (Myanmar)	Pill (Bangladesh)	Orally (Bangladesh)	Herb, Subshrub	
Brazil	Menstrual regulation	Asteraceae	*Tagetes erecta* L.	Fruit	Decoction	Orally	Herb	Bieski et al. (2015)
Brazil	Menstruation	Myrtaceae	*Leptospermum scoparium* J.R. Forst. and G. Forst.	—	—	—	Herb	
Brazil	Menstruation	Aristolochiaceae	*Aristolochia cymbifera* Mart and Zucc	—	—	—	Tree	
Brazil, Thailand	Menstrual disorders(Thailand), Menstrual regulation(Brazil)	Asteraceae	*Artemisia* sp.	Aerial part (Thailand), leaf (Brazil)	Infusion (Brazil)	—	Herb	
China	Menorrhagia	Acanthaceae	*Thunbergia laurifolia* Lindl.	Leaf	—	—	Vine	
Italy	Irregular menstrual cycle	Apiaceae	*Petroselinum crispum* (Mill.) Fuss	Aerial part	Infusion, decoction	Orally	Herb	Motti et al. (2019)
Italy	Irregular menstrual cycle	Asteraceae	*Calendula officinalis* L.	Flower	Infusion	Orally	Herb	
Italy	Irregular menstrual cycle	Pteridaceae	*Adiantum capillus-veneris* L.	Leaf	Decoction	Orally	Herb	
Italy	Irregular menstrual cycle	Rosaceae	*Rubus fruticosus* L.	Leaf	Decoction	Orally	Vine	
Italy	Irregular menstrual cycle	Rosaceae	*Rubus ulmifolius* Schott.	Leaf	Infusion	Orally	Vine	
Italy	Irregular menstruation	Lamiaceae	*Marrubium vulgare* L.	Flower	Macerate	Orally	Herb	
Italy	Menorrhagia	Lamiaceae	*Lamium album* L.	Aerial part	Decoction	Orally	Herb	
Italy	Menorrhagia	Asteraceae	*Bellis perennis* L.	Flower	Infusion	Orally	Herb	
Italy	Menorrhagia	Polygonaceae	*Polygonum hydropiper* L.	Whole plant	Infusion	Orally	Herb	
Italy	Menorrhagia	Rosaceae	*Prunus spinosa* L.	Bark, Root	Decoction	Orally	Shrub	
Italy	Menorrhagia	Urticaceae	*Urtica dioica* L.	Aerial part	Raw	Orally	Herb	
Italy	Oligomenorrhea	Aceraceae	*Acer campestre* L.	Bark	Decoction	Orally	Tree	
Italy	Oligomenorrhea	Sapindaceae	*Acer opalus subsp. obtusatum* (Waldst. and Kit. ex Willd.) Gams	Bark	Decoction	Orally	Tree	
Italy, Korea	Irregular menstrual cycle, menorrhagia(Italy), menstrual disorders(Korea)	Brassicaceae	*Capsella bursa-pastoris* (L.) Medik.	Aerial part, whole plant(Italy),sprout(Korea)	Raw, decoction, infusion	Topically, Orally	Herb	
Italy, Pakistan	Menorrhagia (Italy), Menstrual disorder(Pakistan)	Asteraceae	*Achillea millefolium* L.	Leaf, flower(Italy), Whole plant(Pakistan)	Decoction, infusion,tincture(Italy)	Orally (Italy)	Herb	
Korea	Irregular menstruation	Asclepiadaceae	*Cynanchum wilfordii* (Maxim.) Hemsl.	Root	Brewing	Orally	Vine	Kim et al. (2006), Kim and Song (2011)
Korea	Irregular menstruation	Asteraceae	*Leonurus japonicus* Houtt.	Leaf	Infusion	Orally	Herb	
Korea	Irregular Menstruation	Celastraceae	*Celastrus orbiculatus* Thunb.	Fruit	Powder, Brewing	Orally	Shrub	
Korea	Irregular Menstruation	Malvaceae	*Althaea rosea* (Linn.) Cavan.	Root	Infusion, Decoction	Orally	Herb	
Korea	Irregular Menstruation	Paeoniaceae	*Paeonia lactiflora* Pall.	Root	Decoction	Orally	Herb	
Korea	Irregular Menstruation	Plantaginaceae	*Plantago asiatica* L.	Root	Decoction	Orally	Herb	
Korea	Irregular Menstruation	Rosaceae	*Sanguisorba officinalis* L.	Root	Infusion	Orally	Herb	
Korea	Menstrual disorder	Asteraceae	*Artemisia selengensis* Turcz. ex Bess	Leaf, root	—	—	Herb	
Korea	Menstrual disorder	Brassicaceae	*Cardamine flexuosa* With.	Root	—	—	Herb	
Korea	Menstrual disorder, menstrual pain	Asteraceae	*Artemisia princeps Pamp*. var. *orientalis* (Pampan.) Hara	Leaf	—	—	Herb	
Malay Peninsula	Irregular menstruation	Clusiaceae	*Garcinia mangostana* L.	root	Decoction	Orally	Tree	Ling and Ng (1998)
Malaysia	Abnormal menstruation	Asteraceae	*Artemisia argyi* Levl. et Vant.	Leaf	—	—	Herb	
Malaysia	Emmenagogue	Bromeliaceae	*Ananas comosus* (L.) Merr.	—	—	—	Herb	
Malaysia	Emmenagogue	Poaceae/Gramineae	*Cymbopogon nardus* (L.) Rendle	—	—	—	Herb	
Malaysia	Emmenagogue	Poaceae/Gramineae	*Cymbopogon citratus* (DC.) Stapf	Stem	—	—	Herb	
Malaysia	Emmenagogue	Zingiberaceae	*Curcuma xanthorrhiza* D.Dietr.	—	—	—	Herb	
Malaysia	Irregular menstruation	Zingiberaceae	*Curcuma longa* L.	—	—	—	Herb	
Malaysia	Menstrual Disorders (MDs)	Lamiaceae	*Leonurus artemisia* (Laur.) S. Y. Hu F	Aerial part	—	—	Herb	
Malaysia	Menstrual pains	Phyllanthaceae	*Antidesma velutinosum* Blume	Decoction	—	—	Shrub, tree	
Malaysia	Oligomenorrhoea	Arecaceae/Palmae	*Areca catechu* L.	-	—	—	Tree	
Malaysia, Myanmar	Emmenagogue (Malaysia), regulate menstruation(Myanmar)	Caricaceae	*Carica papaya* L.	Root (Myanmar)	—	—	Tree	
Malaysia, Myanmar	Emmenagogue, oligomenorrhoea	Rubiaceae	*Morinda citrifolia* L.	Leaf, Fruit(Myanmar)	—	—	Shrub, Tree	
Malaysia, Upper Amazon region	Emmenagogue (Malaysia), Regulate menstruation	Myrtaceae	*Psidium guajava* L.	Flower (Upper Amazon region)	—	—	Shrub, Tree	DeFilipps and Krupnick (2018)
Myanmar	Arrested menstruation	Moringaceae	*Moringa oleifera* Lam.	Leaf	Root, bark with warm milk, leaf with decoction	Orally	Shrub	
Myanmar	Menorrhagia, menstrual problems	Apiaceae	*Daucus carota* L.	Root	—	Orally	Herb	
Myanmar, India	Menstrual disorders (Myanmar), Amenorrhea, dysmenorrhea, emmenogogue, oligomenorrhoea, (India)	Xanthorrhoeaceae	*Aloe vera* (L.) Burm.f.	Whole plant, pulp, root, leaf (India)	Juice (India)	Orally (India)	Herb	
Myanmar, India	Oligomenorrhoea	Aristolochiaceae	*Aristolochia indica* L.	Leaf, root	Powder + water (India)	Orally (India)	Herb	
Myanmar, Thailand	Menstrual disorders	Myristicaceae	*Myristica fragrans* Houtt.	Aril of fruit and seed (Thailand)	—	—	Tree	
Myanmar, Thailand, Vietnam	Emmenagogue (Thailand), menorrhagia (Myanmar), menstrual disorders (Vietnam)	Apocynaceae	*Catharanthus roseus* (L.) G.Don	Root (Thailand, Vietnam), leaf (Myanmar)	Aqueous extract (Myanmar)	Orally (Myanmar)	Tree	
Nepal	Menstrual problems	Abaceae	*Arisaema concinnum* Schott	Rhizome	Decoction	Orally	Herb	Malla et al. (2015)
Nepal	Menstrual problems	Anacardiaceae	*Rhus javanica* L.	Fruit	Powder	Orally	Tree	
Nepal	Menstrual problems	Cucurbitaceae	*Solena amplexicaulis* (Lam.) Gandhi.	Root	Decoction	Orally	Herb	
Nepal	Menstrual problems	Ericaeae	*Rhododendron arboreum* Sm.	Petals	Raw	Orally	Tree	
Nepal	Menstrual problems	Fabaceae	*Indigofera bracteata* Grah. ex Baker	Leaf	Juice	Orally	Shrub	
Nepal	Menstrual problems	Hypericaceae	*Hypericum cordifolium* Choisy in DC.	Whole plant	Extract	Orally	Shrub	
Nepal	Menstrual problems	Liliaceae	*Paris polyphylla* Sm.	Rhizome	Juice	Orally	Herb	
Nepal	Menstrual problems	Polygonaceae	*Rheum australe* D. Don.	Rhizome	Juice	Orally	Herb	
Nepal	Menstrual problems	Rhamnaceae	*Zizyphus mauritiana* Lam.	Root	Decoction	Orally	Tree	
Nepal	Menstrual problems	Rosaceae	*Fragaria nubicola* Lindley ex Lacaita.	Whole plant	Juice	Orally	Herb	
Nepal	Menstrual problems	Rutaceae	*Murraya paniculata* (L.) Jack.	Leaf	Decoction	Orally	Shrub	
Nepal	Menstrual problems	Saxifragaceae	*Astilbe rivularis* Buch.-Ham. ex D. Don.	Rhizome	Juice	Orally	Shrub	
Nepal	Menstrual problems	Scrophulariaceae	*Hemiphragma heterophyllum* Wall.	Whole plant	Juice	Orally	Herb	
Pakistan	Excessive menstruation	Amaranthaceae	*Amaranthus viridis* L.	Whole plant, root	—	Topically	Herb	Aziz et al. (2018), Tariq et al. (2018)
Pakistan	Irregular menstruation	Asteraceae	*Calendula arvensis* M. Bieb.	Flower	Infusion	—	Herb	
Pakistan	Irregular menstruation	Geraniaceae	*Geranium wallichianum* D. Don ex Sweet.	Root	Powder + milk	—	Herb	
Pakistan	Irregular menstruation	Hypericaceae	*Hypericum perforatum* L.	Fruit, shoot	Infusion	—	Herb	
Pakistan	Irregular menstruation	Rosaceae	*Prunus domestica* L.	Fruit	—	Orally	Tree	
Pakistan	Irregular menstruation, oligomenorrhea	Apiaceae	*Foeniculum vulgare* Mill.	Leaf, fruit (Irregular menstruation), whole plant (oligoenorrhea)	Orally (Irregular menstruation)	Orally (Irregular menstruation)	Herb	
Pakistan	Menorrhagia	Poaceae/Gramineae	*Desmostachya bipinnata* (L.) Stapf	Leaf	—	—	Herb	
Pakistan	Menstrual disorder	Anacardiaceae	*Schinus molle* L.	Bark, leaf, fruit	—	—	Tree	
Pakistan	Oligomenorrhea	Apiaceae	*Trachyspermum triradiatum Wolff*	Seed	—	—	Herb	
Pakistan	Oligomenorrhea	Fabaceae	*Acacia nilotica* (L.) Delile	Bark	—	Topically	Tree	
Pakistan	Regulate the menses	Apiaceae	*Ammi visnaga* (L.) Lam.	Fruit	Infusion	—	Herb	
Pakistan	Regulate the menses	Apiaceae	*Bupleurum falcatum* L.	Whole plant	Decoction	Orally	Herb	
Pakistan	Regulate the menses	Apiaceae	*Heracleum candicans* Wall. ex DC.	Root	Powder	—	Herb	
Pakistan	Stimulate menstrual flow	Asteraceae	*Carthamus oxycantha M.Bieb.*	Flower, seed	—	—	Herb	
Pakistan	Stimulate menstruation	Asteraceae	*Cichorium intybus* L.	Leaf, root	Decoction	—	Herb	
Pakistan	Irregular menstruation	Zingiberaceae	*Zingiber officinale* Roscoe	Root	Powder + water or milk	—	Herb	
Taiwan	Irregular menstruation	Zingiberaceae	*Elettaria cardamomum* (L.) Maton.	Seed	—	—	Herb	
Taiwan	stimulate menstruation	Zingiberaceae	*Zingiber montanum* (J. Koenig) Link ex A.Dietr*.*	Whole plant	—	—	Herb	
Taiwan	Irregular menstruation	Zingiberaceae	*Curcuma zedoaria* (Christm.) Roscoe	Rhizome (Thailand)	—	—	Herb	
Vietnam	Menstrual disorders	Apiaceae	*Centella asiatica* (L.) Urb.	Whole plant	—	—	Herb	Kurian (2012)
Vietnam	Menstrual disorders	Apocynaceae	*Alstonia scholaris* (L.) R. Br.	Bark, leaf, latex	—	—	Tree	
Vietnam	Menstrual disorders	Cyperaceae	*Cyperus rotundus* L.	Root, Rhizome	—	—	Herb	

This section contains a total of 96 families and 287 species of plants from 15 countries and regions used for the treatment of MDs. Fabaceae is very common some plants have a record in treating MDs in more than two countries. There are three ways to use *Punica granatum* L. to treat menorrhagia in Iran: vaginal suppository, lotion, and balm (marham). In India, its raw dried peels are used for menorrhagia. Myanmar women use their leaves and roots to make decoctions. Women in Myanmar and Thailand use garlic (*Allium sativum* L.) bulbs to regulate menstruation.

## 4 Herbal Medicine Products

The utilization of herbal medicine is increasing every year, which is reflected in the statistics of various countries. A study of participants from 23 countries reveals that 28.9% reported the use of herbal medicines in pregnancy (Kennedy et al., 2013). Approximately 35% of adults in the United States reported current use of herbal medicine (Rashrash et al., 2017). The sale of herbal medicines is growing by 20% annually and forms are the largest growth area in retail pharmacy. The sales of such products have moved from specialty stores to mainline shopping environments (Flanagan, 2001). The popularity of herbal medicine has spawned many health care industries, which have many herbal products on the market for menstrual problems. Table 6 lists some herbal products from various countries.

**TABLE 6:** Herbal medicine products.

Country/region	Premenstrual syndrome	Dysmenorrhea	Amenorrhea	Menstrual disorders
America	Chasteberry Extract (*Vitex agnus-castus* L.), Rose Otto Essential Oil, Chasteberry and Dong Quai Women’s Monthly Support Supplement		Ginger powder (*Zingiber officinale* Roscoe)	
Australia	Vitex Agnus-Castus, Evening Primrose (*Oenothera biennis* L.), St John’s Wort (*Hypericum perforatum* L.)		Chamomile Tea (*Matricaria chamomilla* L.), Yarrow Tea (*Achillea millefolium* L.)	
China	ShuerjingKeli	Yuanhu Zhitong tablet, Tongjing Wan, Tongjingbao Keli	Fuke Tongjing Wan, Extract of *Rosa chinensis* Jacq.	Angelica Liquid Extract, Danggui Yangxue Wan, Danggui Tiaojing Keli, Fuke Tiaojing Pian, Tiaojing Huoxue Jiaonang, Erzhi Wan
India	Aloe vitals capsules of Planet Ayurveda, Valerian tea	German Chamomile oil (*Matricaria chamomilla* L.), Essential oil of fennel (*Foeniculum vulgare* Mill.), Sweet Marjoram oil (*Origanum majorana* L.), Lavender oil (*Lavandula angustifolia* Mill.), Clary Sage oil (Salvia sclarea L.), Coriander (Coriandrum sativum L.)		
Iran	Saffron (*Crocus sativus* L.)	Chamomile tea (*Matricaria chamomilla* L.), Ginger (*Zingiber officinale* Roscoe), Mint tea (*Mentha longifolia* L.)		

**FIGURE 1:**
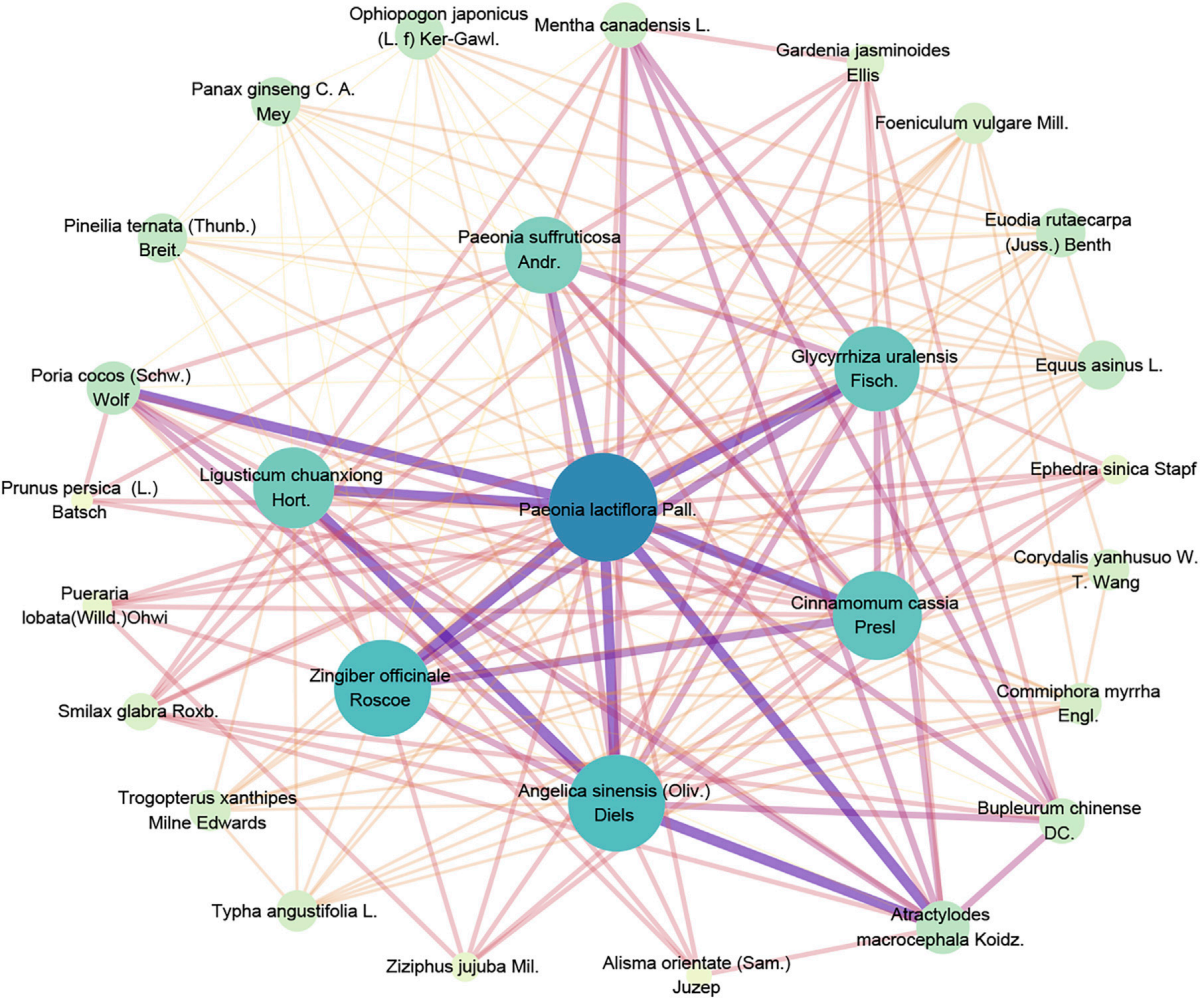
Network diagram of compatibility law of Traditional Chinese Medicine.

Compound Recipes are a major feature of herbal medicine in the treatment of MD. According to the most commonly used traditional Chinese medicine compound (TCMC) in MD showed in Table 1 as an example, there are seven formulae including nonredundant 28 herbs, which totally occurs 57 times, the main herb-pairs and compatibility laws of traditional Chinese medicine prescriptions are listed in Figure 1 as a network diagram obtained by the association rule algorithm-Apriori algorithm.

According to the network, *Paeonia lactiflora* Pall. was in the central with the most relationship with other herbs; *Angelica sinensis* (Oliv.) Diels., *Zingiber officinale* Roscoe., *Cinnamomum cassia* Presl*.*, *Glycyrrhiza uralensis* Fisch., *Ligusticum chuanxiong* Hort., *Paeonia suffruticosa* Andr., *Poria cocos* (Schw.) Wolf., and *Atractylodes macrocephala* Koidz. within the second core layer, with extensive compatibility with other herbs, they are all the backbone medication of TCMC in treating MD.

Among them, according to the theory of TCMC, the Bupleurum-white peony drug pair can regulate menstruation and relieve pain and is also a frequently used drug pair for the treatment of various gynecological diseases. Ginger functions as warming menstruation and is most widely used in TCMC prescriptions for gynecological diseases. It can also be used as a “guiding herb” (which acts an ingredient added to enhance the efficacy of a dose of medicine), which is of great significance to improve the accuracy and efficacy of the medication. Licorice is commonly used in TCMC. It also plays a role in reconciling the medicinal properties and improving the taste. As such, it has a wide range of applications. Licorice can also play a role in pain relief, which is very beneficial in the relieving gynecological disease symptoms such as dysmenorrhea. Angelica is the holy medicine used in gynecology, Chuanxiong is the holy medicine used to activate blood stasis, and cinnamon functions as warming meridians, and atractylodes functions as drying dampness, invigorating the spleen, and relieving pain, which is of great significance for eliminating the cause of the disease.

As a creative contribution of CHM, the compound medicine experience of CHM has affected the traditional medical development of many countries in East Asia, and it will also provide a reference for the development of other ethnic medicines in the future.

## 5 Discussion and Conclusion

In this study, we screened 93 literatures on the topic of MD, among which a review “Medicinal plants used for menstrual disorders in Latin America, the Caribbean, sub-Saharan Africa, South and Southeast Asia and their uterine properties: A review” (van Andel et al., 2014) is similar on this theme, it mainly focuses on MDs and pay more attention on adverse reactions of related herbs. In our study, we systematically analyzed the herbal medicines used in countries and regions in treating different MD, compared the medication common characters and differences, and analyzed the compatibility law of classical prescriptions. As we know, our review is an unprecedented work on comprehensive analysis traditional herbs for MD globally.

Among the herbs used to treat MD, we analyzed 130 families and 571 species of plants used by women from different countries and regions. Among them, 451 are herbs, 178 are trees, 72 are shrubs, 21 are vines, five are climbers and 37 are of other types. The five main families are Asteraceae, Lamiaceae, Apiaceae, Fabaceae, and Zingiberaceae, while the five frequently used plants are *Zingiber officinale* Roscoe. (Ginger), *Ruta graveolens* L. (Common rue), *Angelica sinensis* (Oliv.) Diels (*Angelica sinensis*), *Foeniculum vulgare* Mill. (Fennel), and *Catharanthus roseus* (L.) G. Don (Catharanthus roseus). The general dosage of these herbs is 3–15 g.

Among them, some herbal medicines can treat three MD concurrently such as: *Angelica sinensis* (Oliv.) Diels (Angelica sinensis), *Foeniculum vulgare* Mill *Ligusticum chuanxiong* Hort., *Cyperus rotundus* L., *Spatholobus suberectus* Dunn, *Leonurus japonicus* Houtt., *Salvia miltiorrhiza* Bge., *Prunus persica* (L.) Batsch, *Rosa chinensis* Jacq., *Curcuma longa* L. while some herbal medicines that can treat two MD concurrently such as: *Ruta graveolens* L., *Cinnamomum cassia* (L.) J.Presl, *Sargentodoxa cuneata* (Oliv.) Rehd. et Wils., *Corydalis yanhusuo* W. T.Wang, *Zingiber officinale* Roscoe.

**FIGURE 2:**
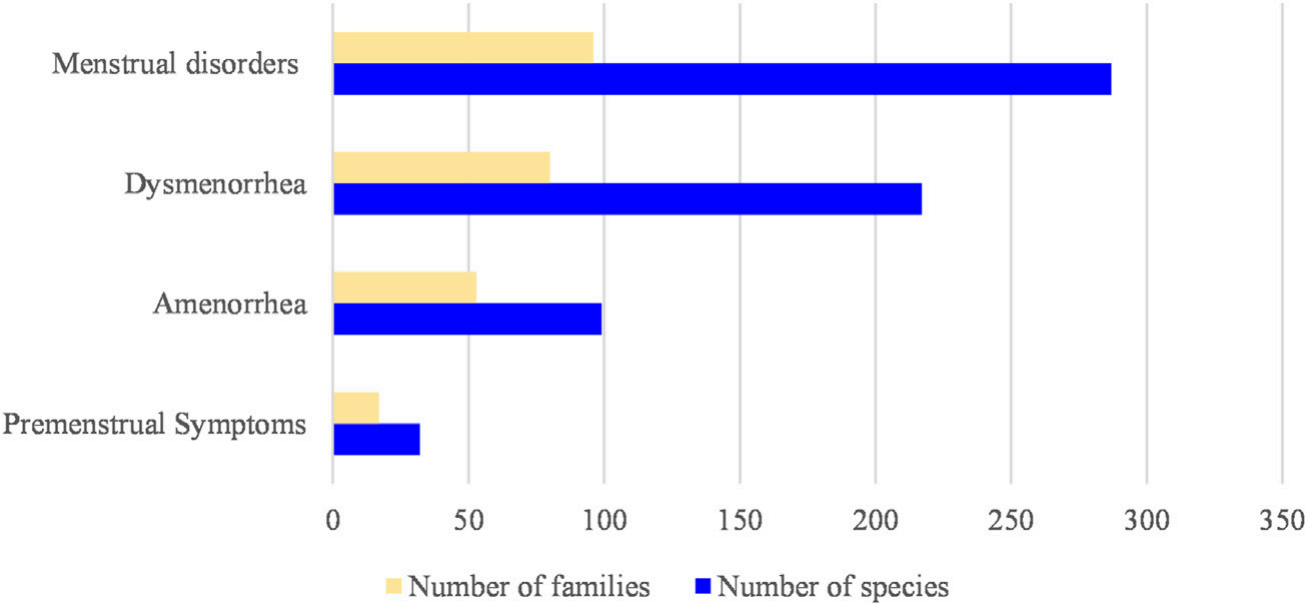
The number of families and species of plants corresponding to each disease type.

The number of families and species of plants corresponding to each disease type is listed in Figure 2. Most types of herbs treat MDs, for there are different symptom categories in MDs; however, considering a single symptom, most types of herbs treat dysmenorrhea. The number of herbs in treating PMS is the least in the four categories of MD, mainly because of PMS was proposed the latest.

**FIGURE 3:**
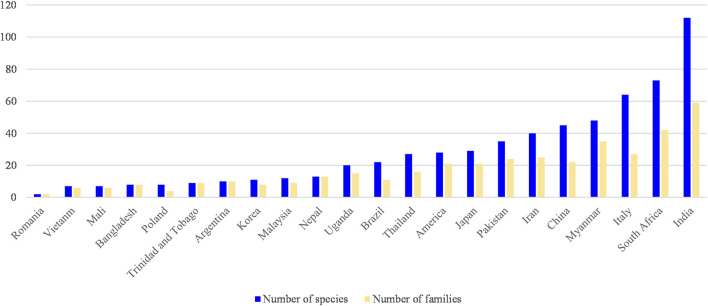
The number of families and species of plants corresponding to each country.

The number of families and species of plants corresponding to each country and region is listed in Figure 3, which shows that India has the most types of individual herbs used to treat MD. The selected literature indicted that India has more medical plant species resources and medication experience in MD, and it was similar for South Africa, Italy and Myanmar, for some literatures specifically collected and summarized herbs in treating MD in these countries or regions, but the application experience and pharmacological differences of many related plants need further research. In China, there are abundant medication experiences in treating MD, but the number of herbs was not increased indefinitely in a long application history, chiefly due to the concept of combined medication in CHM to solve many complex disease problems.

**FIGURE 4:**
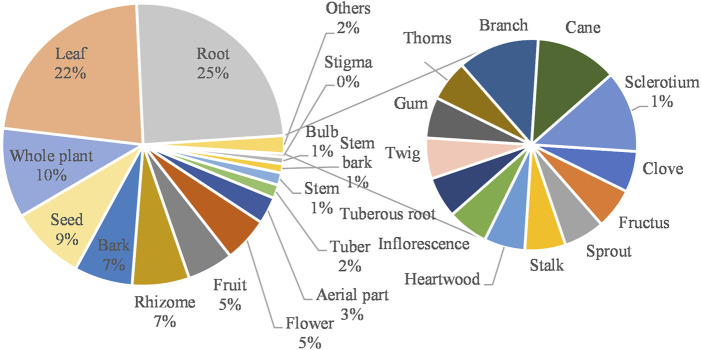
Corresponding proportion of plant medicinal parts.

**FIGURE 5:**
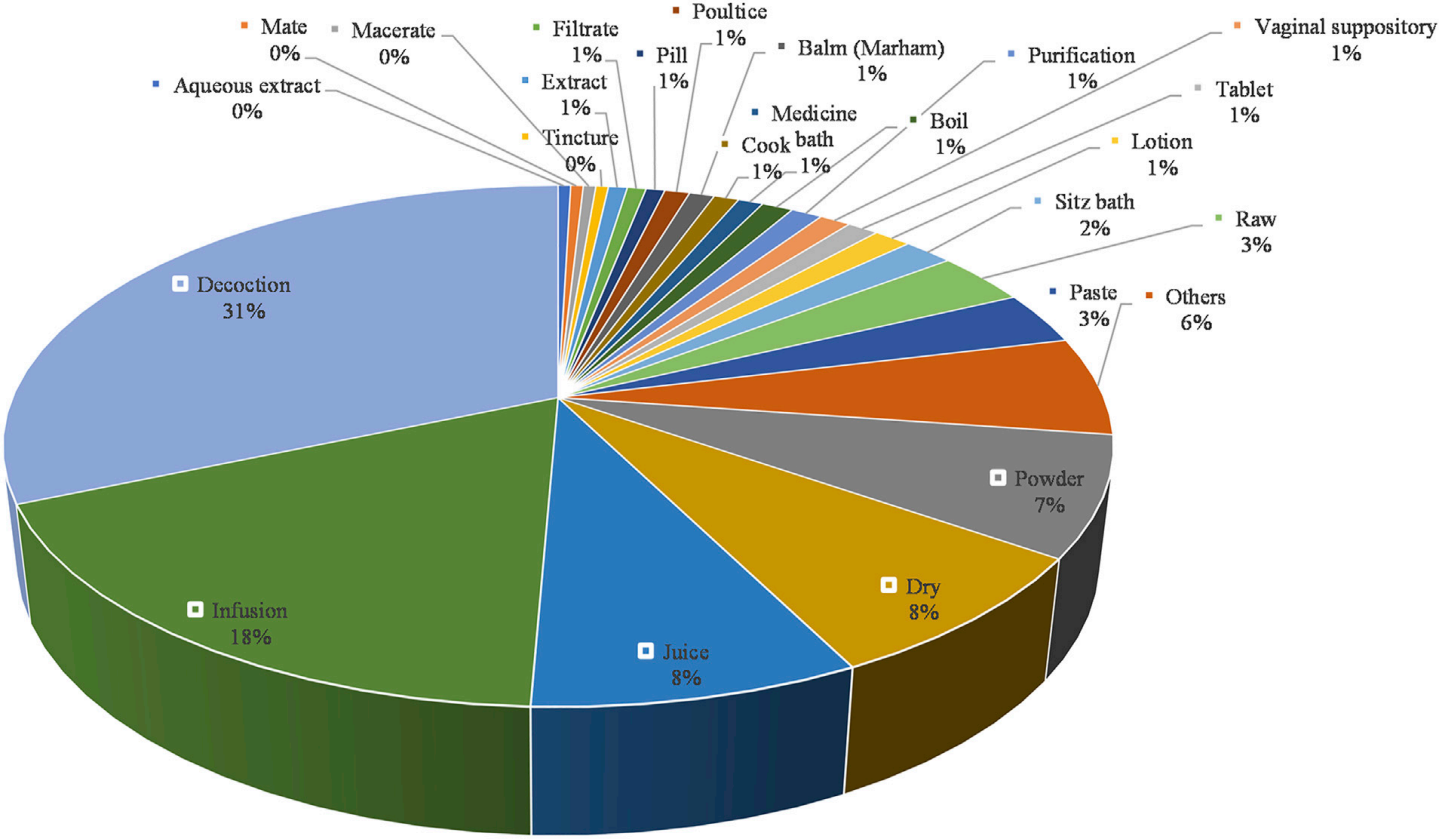
Proportion of plant dosage forms: Others refer to dosage forms that appear only once.

The usage of medicinal parts is listed in Figure 4, which presents the most commonly used parts which are root, leaf, whole plant, seed, bark, rhizome, fruit, flower, and aerial part. The usage of all the herbal medicines mentioned in the article is listed in Figure 5, which illustrates the three most commonly used preparations are decoction, infusion, and juice. Among the recorded routes of administration, 263 are oral administration and 50 are topical administration. The most commonly used type of dosage forms include decoction, infusion, juice, powder, dry, raw, paste, sitz bath, lotion, balm (Marham), and vaginal suppository. The most frequently used ingredients to match the dosage form are honey, milk, sugar, and other excipients, which may improve the taste of herbal medicines, moisturize the intestines and improve patient compliance for usage.

Among the recorded plants, many have culinary use in the population, such as *Coriandrum sativum* L. (Coriander Herb), *Cuminum cyminum* L. (Cumin), *Crocus sativus* L. (Saffron), *Mentha pulegium* L. (Pennyroyal), *Mentha x piperita* L. (Peppermint), *Cinnamomum verum* J. Presl (Ceylon cinnamon), *Zingiber officinale* Roscoe (Ginger), *Allium sativum* L. (Garlic), *Foeniculum vulgare* Mill. (Fennel), *Capsella bursa-pastoris* (Linn.) Medic. (Shepherd’s purse), *Raphanus sativus* L. (Radish), and *Apium graveolens* L. (Celery). Edible herbs are used in a wide range of countries or regions or have multiple effects on MD. This may mean that the edibility of herbal medicine has expanded its promotion. As such, it can be spread and applied in many countries and regions.

Some herbs have the same purpose in different countries. For example, ginger (*Zingiber officinale* Roscoe) is used to treat dysmenorrhea in India, Iran, and Malaysia; and the coriander herb (*Coriandrum sativum* L.) can treat menorrhagia in both India and Iran. Some herbs have different uses in different countries, such as Catharanthus roseus (*Catharanthus roseus* (L.) G. Don) is used to treat dysmenorrhea in India, amenorrhea in Myanmar, MDs in Vietnam, and as an emmenagogue in Thailand. East Asia, Pakistan, Malaysia, and some other places have recorded using fresh herbs. This may be because local therapists believe that fresh herbs are more effective (Pharmacopoeia Commission, 2020).

The application of some drugs is peculiar to this region. For example, Indian women use *Vitex negundo* L. to treat dysmenorrhea and amenorrhea, while Iranian women often use pomegranate (*Punica granatum* L.) to treat menorrhagia. Chinese women usually use roots of *Angelica sinensis* (Oliv.) Diels. for curing various MDs. The whole plant of *Cirsium souliei* (Franch.) Mattf. is applied to treat menorrhagia and the aerial part of *Lagotis brevituba* Maxim is used for menstrual regulation by Tibetan women (Zhu et al., 2017). Mongols frequently apply the aerial part of *Panzeria alaschanica* Kupr. to treat dysmenorrhea (Lei et al., 2017). As a traditional medicine of Uygur nationality, aerial part of *Saussurea involucrata* (Kar. et Kir.) Sch. -Bip is used for the treatment of excessive leucorrhea, but in CHM, it is used for irregular menstruation.

The sources of information on women’s use of plants come from herbal works, multi-media, the internet, medical education, ancestral medical inheritance, doctor-patient communication, neighborhood introduction, and self-exploration. For example, the elder Red-headed Yao women in China play an important role in the spread and utility of medicinal plants in treating gynecological diseases to young women. They can classify medicinal plants, and remember their functions and methods of disease treatment transferred to them from their previous generation.

East Asia has a long-standing habit of using herbal medicines, while in Western countries, herbal medicines have always been used as complementary and alternative treatments. In CHM, the specific details of the menstrual cycle (duration, volume, the appearance of flow, etc.) are not only the surface phenomena, which hint at the underlying causes of MD such as the dysfunction of the internal organs, disharmony of Qi, Blood and Body Fluids, imbalance of the Ren and Du meridians, and the irregularity of Kidney Essence, but symptomatic and causal treatment is required based on these phenomena (Zhou and Qu, 2009). As mentioned above, affected by the culture of Chinese medicine, Japan and South Korea have similar concepts in the treatment of MD, which is also reflected in their medication.

In conclusion, this study compares and summarizes the use of herbal medicines by women in different countries and regions globally. We hope to enlighten people working in these areas, and provide some insight on women’s medication.

## Abbreviations

CHM, Chinese herbal medicine; JXP, jiawei xiaoyao powder; MD, menstrual diseases; MDs, menstrual disorders; PMS, premenstrual syndrome; TPM, traditional Persian medicine.
